# Salidroside ameliorates Parkinson's disease by inhibiting NLRP3-dependent pyroptosis

**DOI:** 10.18632/aging.103215

**Published:** 2020-05-19

**Authors:** Xue Zhang, Yiming Zhang, Rui Li, Lingpeng Zhu, Buqing Fu, Tianhua Yan

**Affiliations:** 1 Department of Physiology, School of Basic Medicine and Clinical Pharmacy, China Pharmaceutical University, Nanjing 210009, China; 2 Department of Inspection, The Affiliated Jiangsu Province Hospital of Chinese Medicine of Nanjing University of Chinese Medicine, Nanjing 210009, China; 3 Center of Clinical Research, The Affiliated Wuxi People's Hospital of Nanjing Medical University, Wuxi 214023, China

**Keywords:** pyroptosis, NLRP3, salidroside, Parkinson's disease, MPTP

## Abstract

Parkinson's disease (PD) is a common age-related neurodegenerative movement disorder, which is mainly due to the loss of dopaminergic neurons. Pyroptosis is a new programmed cell death characterized by NLR Family Pyrin Domain Containing 3 (NLRP3)-dependent, IL-1β, IL-18 and Gasdermin D. Salidroside (Sal) has been reported to have neuro-protective effect. However, the roles of pyroptosis and Sal on anti-pyroptosis in PD have not been elucidated. In this study, we tested underlying mechanisms of pyroptosis in PD and neuro-protective effects of Sal. We established 1-methyl-4-phenyl-1,2,3,6-tetrahydropyridine (MPTP)-induced C57BL/6J mice and C57BL/10ScNJ (TLR4-deficient mice) *in vivo*, MPTP-induced PC-12 and LPS-induced BV2 *in vitro*. We found that Sal could ameliorate MPTP-induced PD symptoms and reduce the levels of IL-1β, IL-18 and Gasdermin D, which are main hallmarks of pyroptosis. Further study indicated that Sal alleviated PD through inhibiting NLRP3-dependent pyroptosis. In conclusion, pyroptosis plays a key role in PD and Sal protects dopaminergic neurons by inhibiting NLRP3-dependent pyroptosis through: (1) indirectly reducing the production of NLRP3, pro-IL-1β and pro-IL-18 by inhibiting TLR4/MyD88/NF-κB signaling pathways, (2) directly suppressing pyroptosis through inhibiting TXNIP/NLRP3/caspase-1 signaling pathways. These results indicated that inhibiting pyroptosis or administration of Sal could be a novel therapeutic strategy for PD.

## INTRODUCTION

Parkinson's disease (PD) is the second most common neurodegenerative disease after Alzheimer’s disease. It is estimated that about 4.94 million patients suffer from PD in China, accounting for half of the worldwide PD patients by 2030 [1]. PD patients always involve motor deficits including bradykinesia, resting tremor, muscle rigidity, impaired gait and neuropsychiatric disturbances [2, 3]. To date, although symptomatic treatments exist, no current therapies can effectively slow or prevent the progression of PD. Therefore, there is an urgent need to develop an anti-PD agent which not only ameliorates PD, but also has neuro-protective effects. Meanwhile, it is essential to understand the potential mechanisms of PD.

PD is characterized by the accumulation of alpha-synuclein (α-syn) into filamentous aggregates [4, 5]. In addition, the primary pathological feature of PD is the progressive loss of the dopaminergic neurons in the substantia nigra (SN) and striatum, which control motor system [6–8]. Neuronal dopamine (DA) concentration is upregulated by intracellular DA biosynthesis and DA reuptake system. Tyrosine hydroxylase (TH), a key enzyme for DA biosynthesis, is decreased in PD, which is a hallmark in the progression of PD [9, 10]. Interestingly, it was reported that the loss of dopaminergic neurons resulted from apoptosis, necrosis and autophagy in PD [11–13]. However, there is no unified definite theory to explain the cause of loss of dopaminergic neurons. Recent studies have revealed other type of programmed cell death, pyroptosis, which is likely to participate in the process of loss of dopaminergic neurons [14–17]. Cells undergoing pyroptosis share some features with apoptotic cells such as nuclear condensation and chromatin DNA fragmentation [15, 18, 19]. However, the pro-inflammatory nature distinguishes pyroptosis from apoptosis despite the dependency on caspase proteins [19, 20]. Similar to necrosis, pyroptosis is also executed by altering plasma membrane permeability, but pyroptosis exhibits no ion selectivity. The formation of pores during pyroptosis disrupts the balance of ion gradients on both sides of the membrane and leads to water inflow. Cell membrane were ruptured and released intracellular proinflammatory, including IL-1β, IL-18 and HMGB1, which are sufficient to cause a cascade of inflammatory responses, thus pyroptosis is also known as inflammatory “necrosis” [15, 19, 21]. Pyroptosis, a specialized and pro-inflammatory form of programmed cell death, relies on the enzymatic activity of cysteine-dependent aspartate-specific protease (caspase) family [15–17]. Furthermore, as a new discovered pyroptosis executive protein, Gasdermin (GSDM) is a member of conserved proteins, including GSDMA, GSDMB, GSDMC, GSDMD, DFNA5 and DFNB59. Among them, GSDMD is essential in pyroptosis, whose gasdermin-N and -C domains can be cleaved by caspase family [22–24]. Pyroptosis is a critical response of innate immune system and initiated by inflammasome through inflammatory caspase proteins, such as caspase-1, 4, 5 (humans) and caspase-1, 11(mice) [22]. As a part of immune system, inflammasome are multiprotein complexes assembled by pattern recognition receptors (PRRs). There are five types of inflammasome, such as NLRP1 inflammasome, NLRP3 inflammasome, NLRC4 inflammasome, IPAF inflammasome and AIM2 inflammasome. The most well-known inflammasome is the NOD (nucleotide binding oligomerization domain)-like receptors family pyrin domain containing 3 (NLRP3) inflammasome. NLRP3 inflammasome is mainly composed of NLRP3, the signaling adapter apoptosis-associated speck-like protein containing caspases recruitment domain (ASC) and caspaes-1 [25, 26]. Neuro-inflammation is common feature of neurodegenerative pathologies, for example PD [27, 28]. Accumulating evidence suggest that the inflammatory cytokines such as interleukin 18 (IL-18), IL-1β and IL-6 play a vital role in the central nervous system [28, 29]. As reported, the levels of IL-1β and IL-18 are significantly increased in PD patients [29]. Previous studies found that they are two main indicators in pyroptosis [26, 28]. Therefore, pyroptosis may be crucial for regulation of central nervous system (CNS) inflammation in PD. Activation of NLRP3 inflammasome promotes the secretion of IL-1β, IL-18 and the formation of GSDMD pore by activating caspase-1 [24, 29]. Thus, NLRP3-dependent pyroptosis is critical in PD.

**Figure 1:**
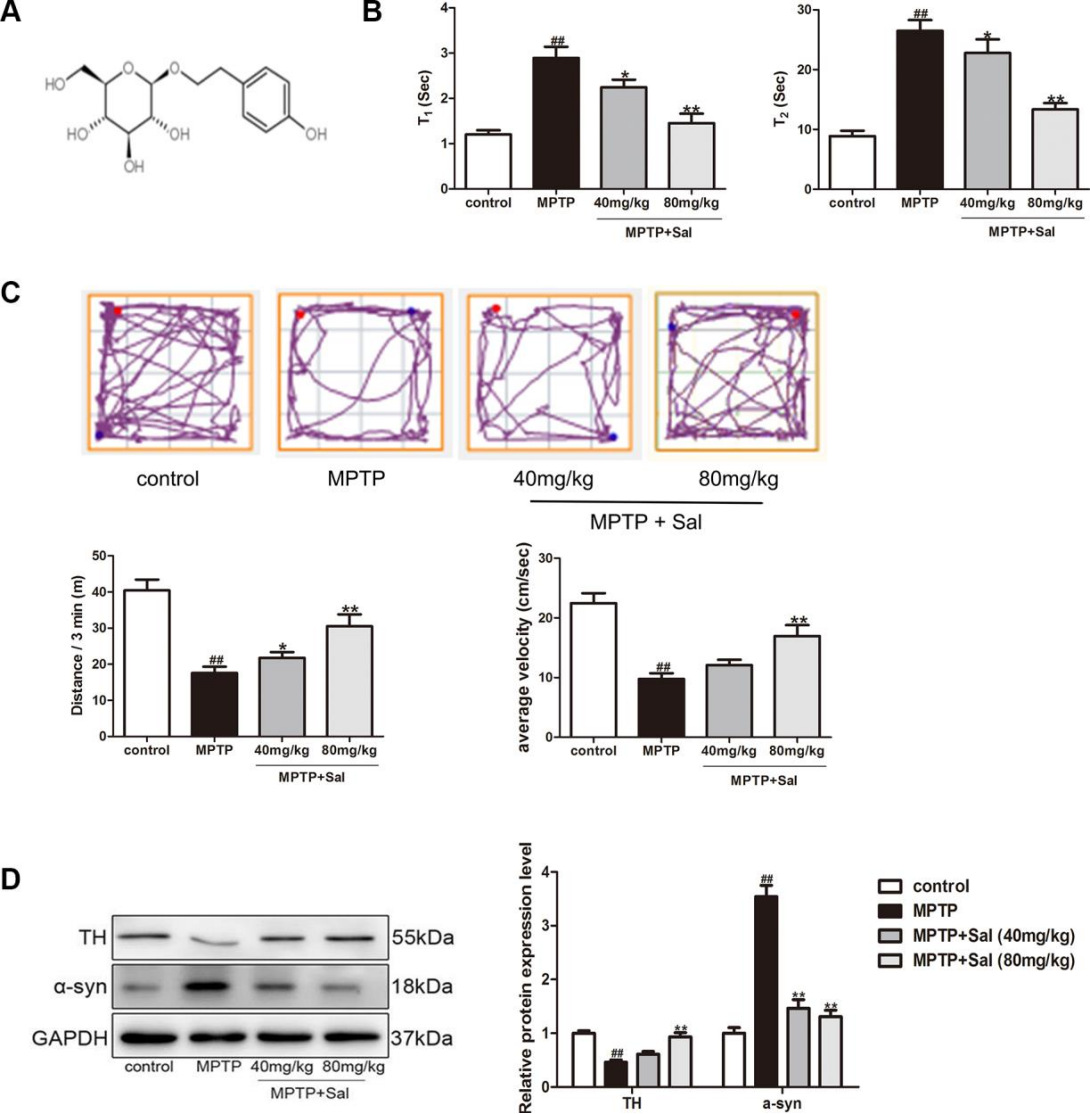
**Sal improved MPTP-induced PD mice.** (**A**) The structure of Sal. (**B**) The performance of Pole test in MPTP-induced mice. The time for mice to turn from upward to downward (T_1_) and to climb down the pole (T_2_) was determined (n = 10). (**C**) The track of mice treated with MPTP with or without Sal, 3 min total traveled distance (Distance/3 min) and average velocity (average velocity / 3 min) (n = 10). (**D**) Western blotting was performed to determine the expression of TH and α-syn in Substantia nigra (SN) and striatum of PD mice (n = 3). All data are represented as mean ± SD. ^#^ P < 0.05, ^##^ P < 0.01 vs. control group, *P < 0.05, **P < 0.01 vs MPTP group.

Salidroside (Sal, 2-(4-phydroxyphenethyl)ethyl-β-D-glucopyranoside, C_14_H_20_O_7_, structure shown in Figure 1A), one of the main bioactive compounds extracted from *Rhodiola rosea L.*, has a wide spectrum of pharmacological effects, such as anti-inflammatory [27], anti-oxidant [30], anti-depressive [31], anti-radiation [32], anti-cancer [33] and cardio-protective [34]. Notably, Sal may act as potential neuro-protective agent through regulating the ROS-NO-related mitochondrial pathways [35]. However, the neuro-protective effects of Sal and its potential mechanisms have remained elusive. Therefore, this study aims to provide a potential new insight into the therapeutic effects of Sal in PD and attempts to explore its molecular mechanisms.

## RESULTS

### Sal improved MPTP-induced PD mice

### Pole test

The mice were subjected to the pole test to evaluate bradykinesia. The results showed that MPTP significantly prolonged the time to orient downward (T_1_) and descend the pole (T_2_) in mice, which indicated MPTP induced mice bradykinesia. The mice treatment with Sal (40, 80 mg/kg,) significantly reversed the MPTP-induced prolongation of T_1_ and T_2_ (Figure 1B).

### Open-field test

We conducted the open-field test to assess spontaneous exploration and emotional response in each group. The results showed the traces pattern of movement in the different experimental groups (Figure 1C). The MPTP group explored the center of the open-field arena significantly less than the control group. The Sal (80 mg/kg) group, although not quite as active as the control group, showed obviously more movement than MPTP group. The MPTP (80 mg/kg) group significantly decreased the distance of locomotor activity and average velocity (P < 0.01). Furthermore, the MPTP mice tended to spend more time on rests during their exploration, compared with the control group. The mice treatment with Sal (40, 80 mg/kg) significantly improved MPTP-induced abnormal spontaneous exploratory behavior.

### Sal improved MPTP-induced brain TH and α-syn expression

**Figure 2:**
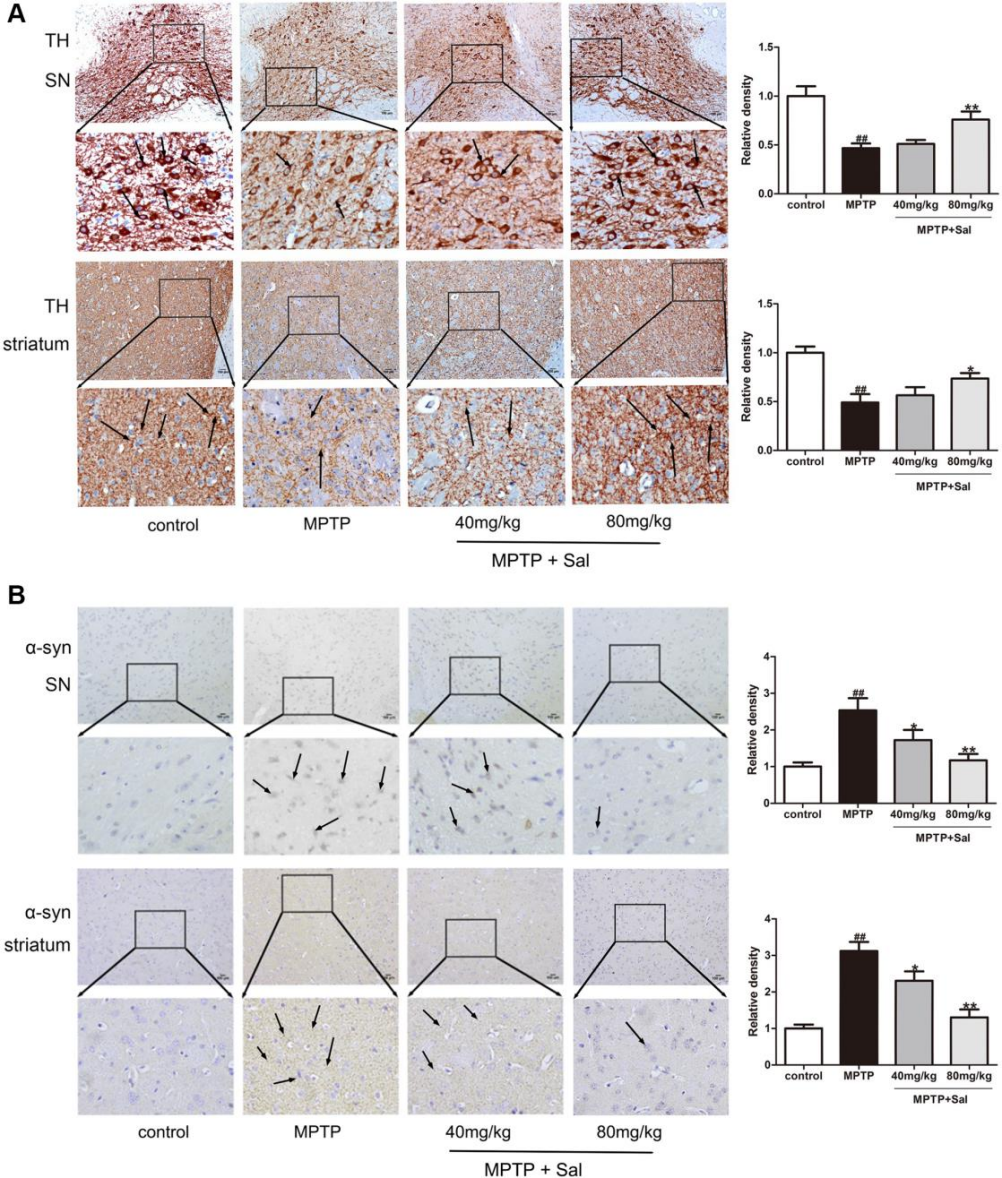
**Sal upregulated TH and downregulated α-syn in PD mice.** Immunochemical staining of TH (**A**) and α-syn (**B**) in SN and striatum and the relative density of related protein in SN and striatum (n = 3). Original magnification: x200. TH neurons in SN and striatum were manually counted by Image J (Abnormal morphological cells were not counted). All data are represented as mean ± SD. ^#^ P < 0.05, ^##^ P < 0.01 vs. control group, *P < 0.05, **P < 0.01 vs MPTP group.

TH is the rate-limiting enzyme in dopamine (DA) synthesis and α-syn is the main component of Lewy body, which are two main characteristic markers in PD patients [36]. To evaluate whether Sal could improve MPTP-induced PD mice, we detected TH and α-syn expression in the SN and striatum by western blotting and immunohistochemistry analysis. Sal up-regulated TH and down-regulated α-syn in PD mice, which was detected by western blotting (Figure 1D). Moreover, Immunohistochemical staining results showed that significant reduction of TH-positive cells in SN and striatum for MPTP group, and Sal significantly improved TH expression in SN and striatum (Figure 2A). The level of α-syn was notably enhanced in MPTP group by immunohistochemistry, whereas these alterations were significantly inhibited by Sal (Figure 2B). The above experiments confirmed that Sal could improve the symptoms of MPTP-induced PD mice.

### Sal alleviated pyroptosis in PD mice

**Figure 3:**
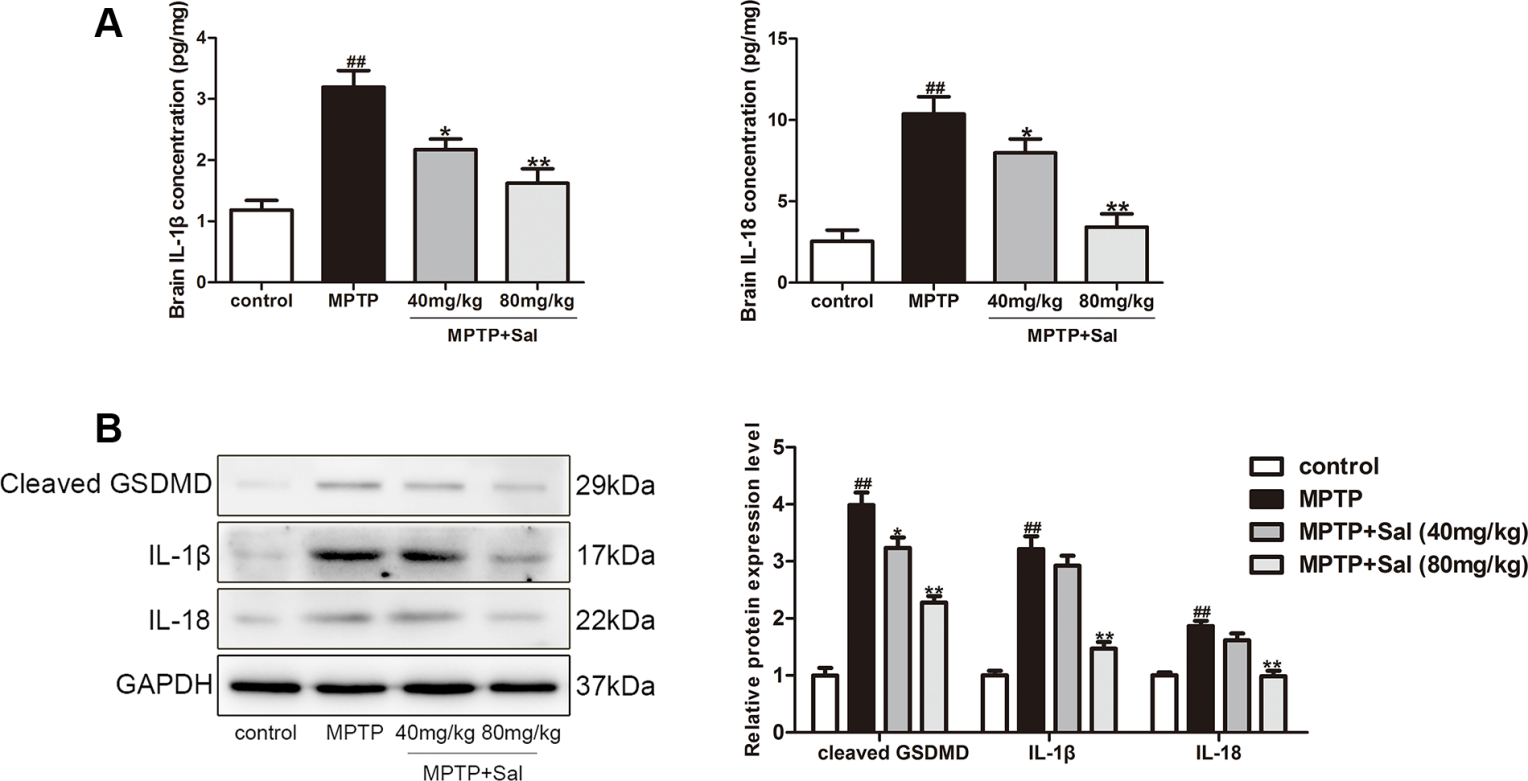
**Sal alleviated pyroptosis in PD mice.** (**A**) Sal inhibited MPTP-induced the increase of IL-1β and IL-18 in SN and striatum of PD mice by enzyme-linked immunosorbent assay (ELISA) kits (n = 6). (**B**) Sal inhibited the expression of cleaved GSDMD, IL-1β and IL-18 in SN and striatum of PD mice by Western blotting. (n = 3). All data are represented as mean ± SD. ^#^ P < 0.05, ^##^ P < 0.01 vs. control group, *P < 0.05, **P < 0.01 vs MPTP group.

To verify the pyroptosis in PD mice, we detected the levels of the key pyroptosis indicators IL-1β and IL-18 by enzyme-linked immunosorbent assay (ELISA) kits in PD mice. As expected, both factors notably increased in the brain of PD mice, which indicated that IL-1β and IL-18 may play an important role in the development of PD mice (Figure 3A). Sal remarkably decreased the levels of IL-1β, IL-18 in PD mice. The activated GSDMD could promote the secretion of IL-1β and IL-18 [22], which can aggravate pyroptosis. In western blotting, IL-1β, IL-18 and cleaved GSDMD significantly increased in the brain of PD mice. Consistent with the ELISA results, Sal remarkably decreased the levels of IL-1β, IL-18 and cleaved GSDMD in PD mice (Figure 3B). These results demonstrated that pyroptosis was widespread in PD mice and Sal could ameliorate pyroptosis.

### Sal inhibited pyroptosis via inhibiting TLR4/MyD88/NF-κB and TXNIP/NLRP3/ Caspase-1 signaling pathways in PD mice

**Figure 4:**
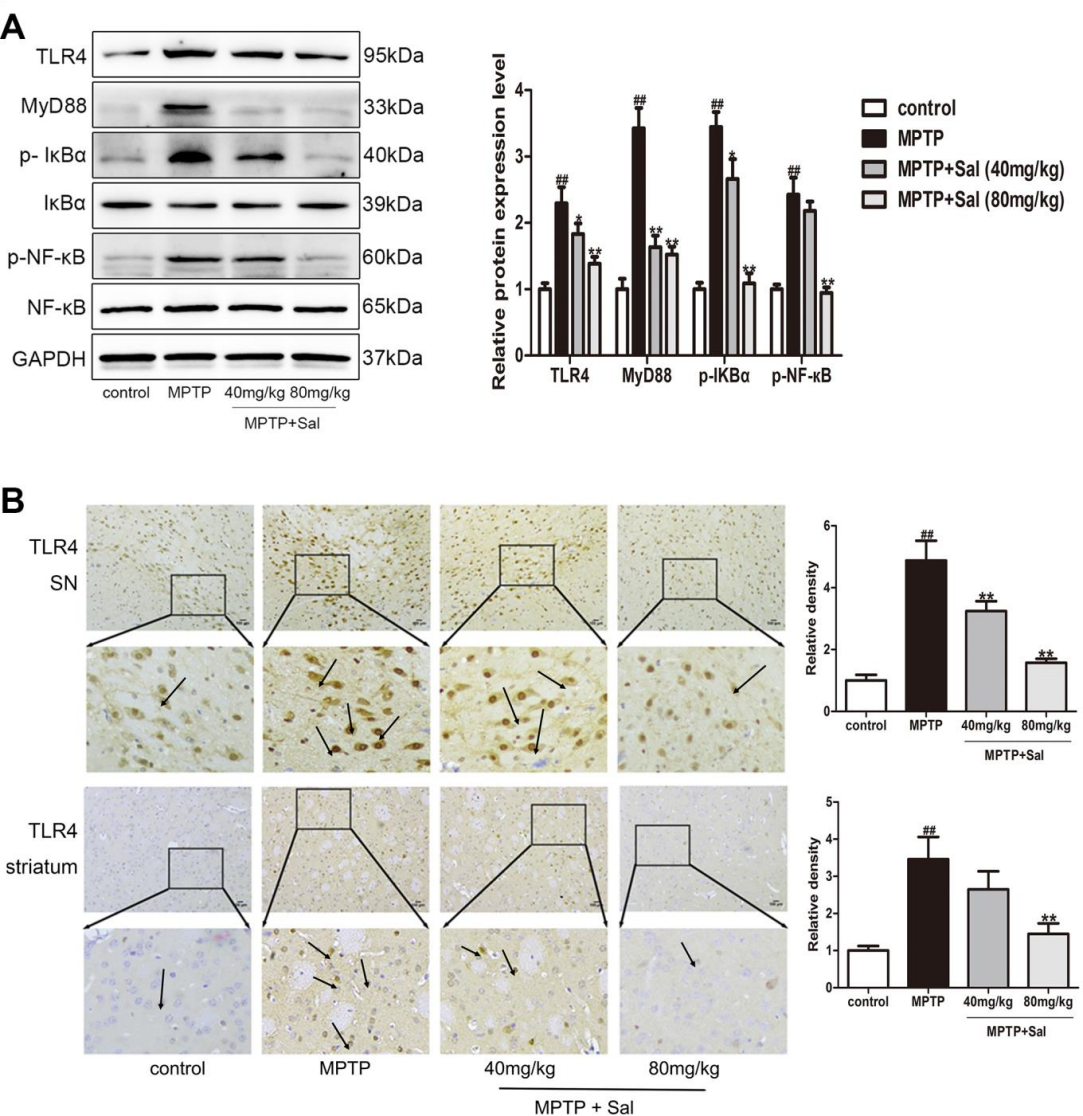
**Sal inhibited pyroptosis via the TLR4/MyD88/NF-κB signaling pathways in PD mice.** (**A**) Sal inhibited TLR4, MyD88, p-IкBα and p-NF-κB in SN and striatum of PD mice by western blotting (n = 3). (**B**) Sal inhibited TLR4 in SN and striatum by immunohistochemical staining. Original magnification: x200. All data are represented as mean ± SD. ^#^ P < 0.05, ^##^ P < 0.01 vs. control group, *P < 0.05, **P < 0.01 vs MPTP group.

**Figure 5:**
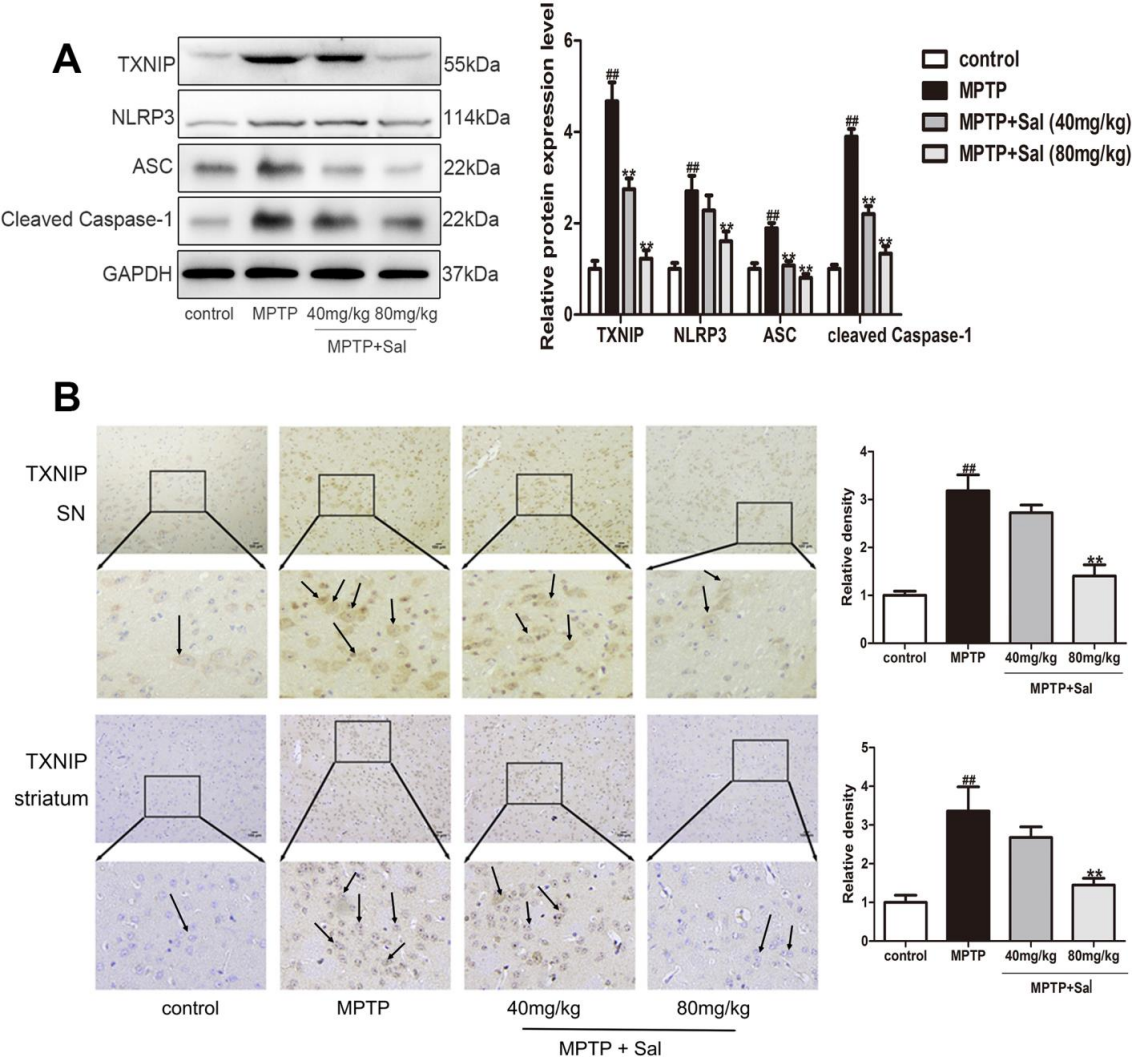
**Sal inhibited pyroptosis via NLRP3/ASC/Caspase-1 signaling pathways in PD mice.** (**A**) Sal inhibited TXNIP, NLRP3, ASC and cleaved Caspase-1 in SN and striatum of PD mice by western blotting (n = 3). (**B**) Sal inhibited TXNIP in SN and striatum by immunohistochemical staining. Original magnification: x200. All data are represented as mean ± SD. ^#^ P < 0.05, ^##^ P < 0.01 vs. control group, *P < 0.05, **P < 0.01 vs MPTP group.

In order to investigate the pyroptosis mechanisms in PD and anti-pyroptosis of Sal, western blot and immunohistochemistry experiments were investigated (Figures 4, 5). In the western blotting analysis, the expressions of TLR4, MyD88, p-IкBα, p-NF-кB, TXNIP, NLRP3, ASC and cleaved Caspase-1 were significantly increased in MPTP-induced PD mice, while Sal significantly reversed these changes (Figures 4A, 5A). Consistently above, the results of the immunohistochemistry experiment showed that Sal treatment significantly suppressed the expressions of TLR4 (Figure 4B) and TXNIP (Figure 5B) in PD mice. The above experiments verified that pyroptosis was associated with the TLR4/MyD88/NF-κB and TXNIP/NLRP3/Caspase-1 signaling pathways and Sal alleviated pyroptosis by inhibiting the above pathways.

### Sal prevented PC-12 cells pyroptosis though inhibiting the TLR4/MyD88/NF-κB and TXNIP/NLRP3/Caspase-1 signaling pathways

**Figure 6:**
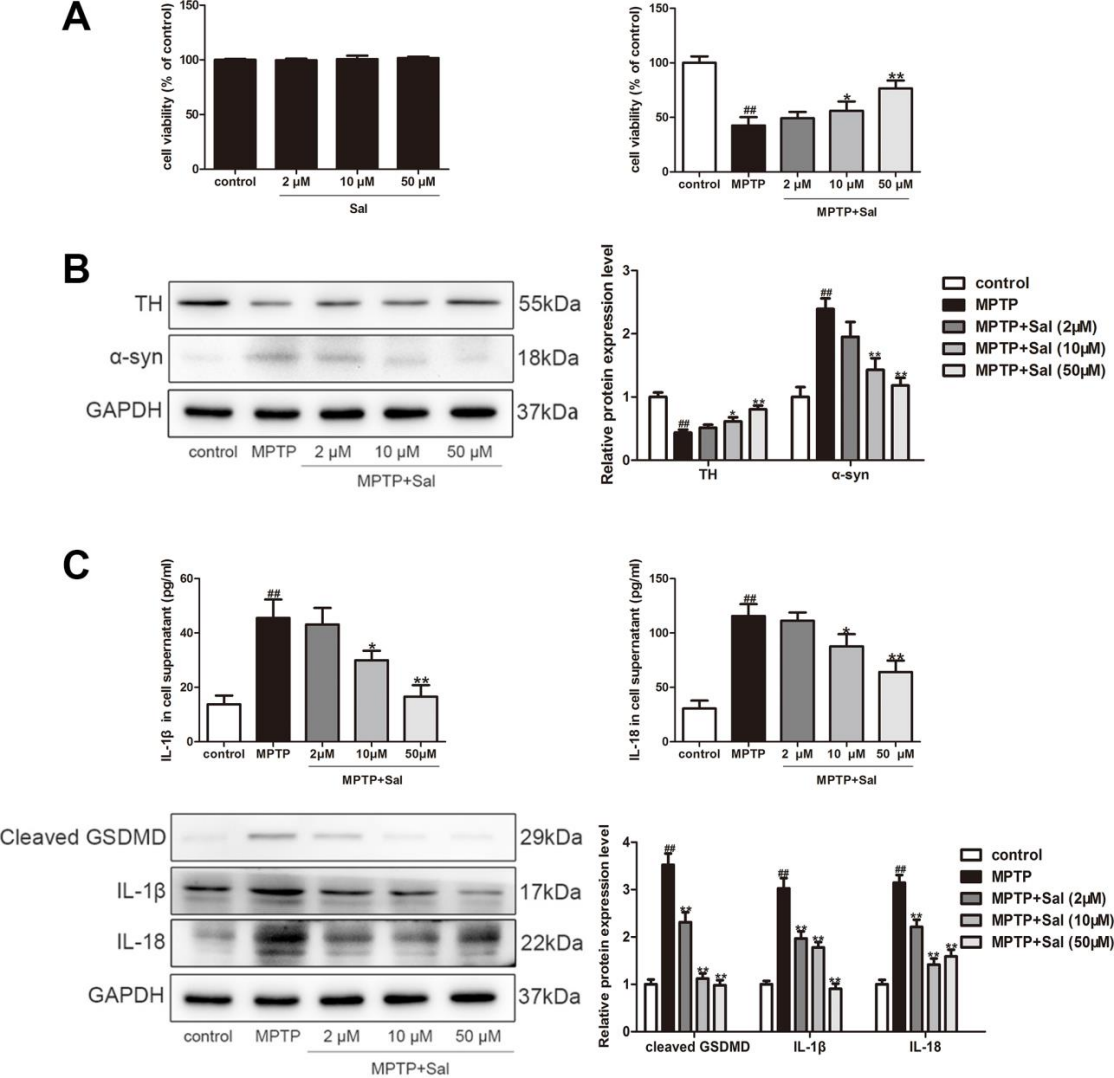
**Sal improved MPTP- induced PC-12 cells.** (**A**) Sal alone does not affect PC-12 cell viability but inhibits MPTP-induced the reduction of PC-12 cell viability. PC-12 cells (1 x 10^4^ cells/well) were exposed to a series concentrations of Sal (2, 10, 50 μM) for 24 h to determined the toxicity of Sal. PC-12 cells were incubated with Sal (2, 10, 50 μM) for 2 h, and then exposed to 500 μM MPTP for 24 h to determined the protective of Sal. The cell viability was measured by cell counting kit-8 (CCK-8) assay. (**B**) Sal inhibited MPTP-induced decreased TH and increased α-syn in PC-12 cells by Western blotting. The cells were incubated with Sal (2, 10, 50 μM) for 2 h, followed by stimulation with MPTP (500 μM) for 24 h. (**C**) Sal inhibited MPTP-induced increased of IL-1β, IL-18 and cleaved GSDMD in PC12 cells. Cells were incubated with Sal (2, 10, 50 μM) for 2 h, followed by stimulation with MPTP (500 μM) for 24 h. The levels of IL-1β and IL-18 in the supernatant were determined by ELISA, and protein of IL-1β, IL-18 and cleaved GSDMD in cells were determined by Western blotting. All data are represented as mean ± SD. ^#^ P < 0.05, ^##^ P < 0.01 vs. control group, *P < 0.05, **P < 0.01 vs MPTP group.

To determine whether Sal effects cell viability, PC-12 cells were exposed to Sal (2, 10, 50 μM) for 24 h. The results showed that Sal (2, 10, 50 μM) treatment did not affect the viability of PC-12 cells by CCK-8 assay. Subsequently, we investigated the effect of Sal on cell viability in MPTP-induced PC-12 cells. We found Sal could significantly reverse the viability of MPTP-induced PC-12 cells (Figure 6A). Furter studies found that MPTP (500 μM) obviously decreased TH and increased α-syn in PC-12 cells, which is consistent with the results *in vivo*, and Sal (2, 10, 50 μM) significantly restored these alterations (Figure 6B). These results showed Sal could prevent α-syn aggregation and increase TH in MPTP-induced PC-12 cells.

**Figure 7:**
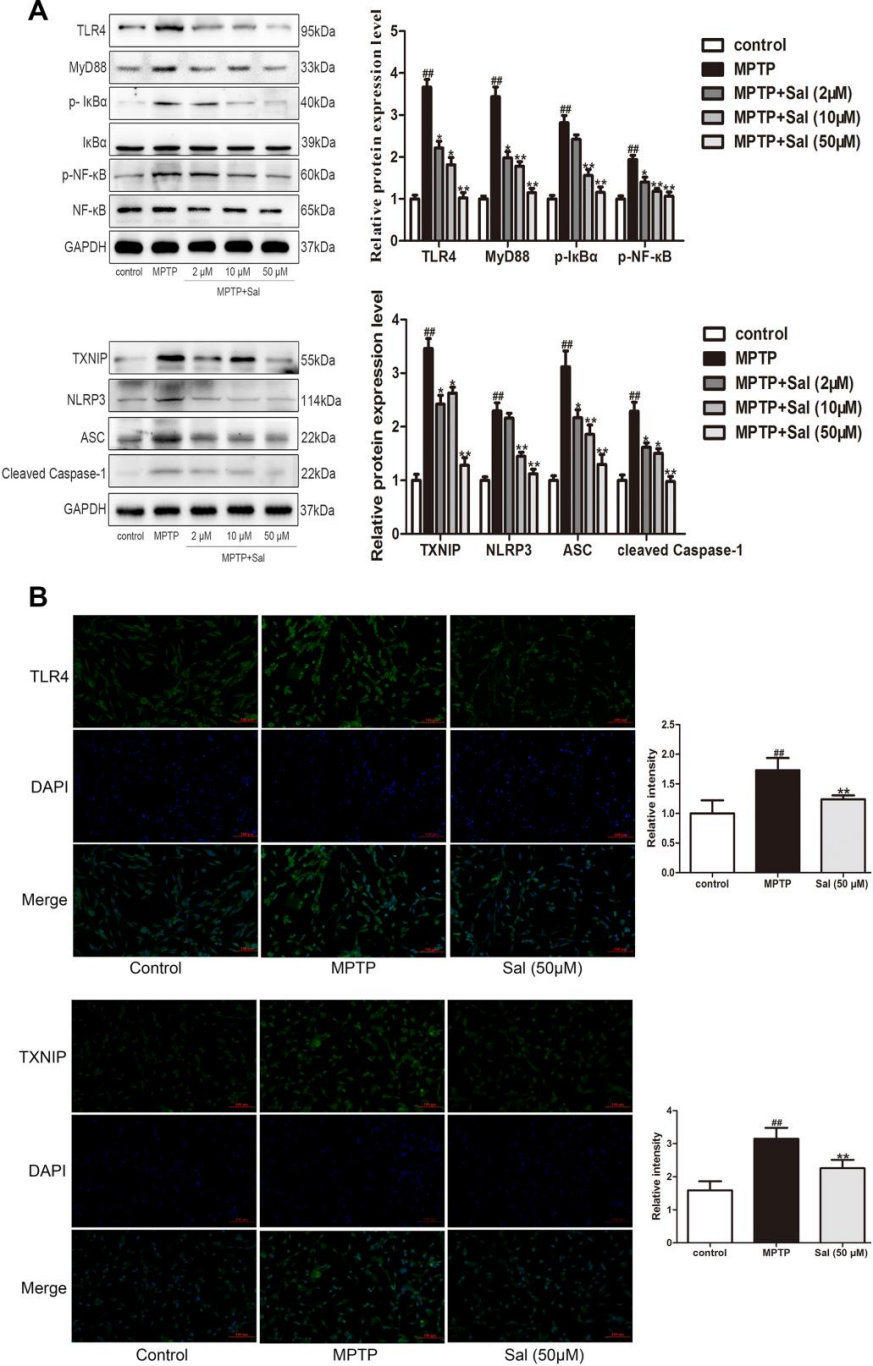
**Sal prevented PC-12 cells pyroptosis through inhibiting the TLR4/MyD88/NF-κB and NLRP3/ASC/Caspase-1 signaling pathways.** (**A**) Sal inhibited MPTP-induced PC-12 cells pyroptosis through TLR4/MyD88/NF-кB and TXNIP/NLRP3/Caspase-1 signaling pathways. Cells were incubated with Sal (2, 10, 50 μM) for 2 h, followed by stimulation with MPTP (500 μM) for 24 h. The protein expressions of TLR4, MyD88, p-IкBα, p-NF-кB, TXNIP, NLRP3, ASC and cleaved Caspase-1 in MPTP-induced PC-12 cells were determined by western blot. (**B**) Sal inhibited MPTP-induced increase of TLR4 and TXNIP by immunofluorescence. Original magnification: x200. All data are represented as mean ± SD. ^#^ P < 0.05, ^##^ P < 0.01 vs. control group, *P < 0.05, **P < 0.01 vs MPTP group.

Consistently, we detected the levels of inflammatory factors IL-1β and IL-18 in the MPTP-induced PC-12 cells (Figure 6C). The findings, consistent with the results *in vivo*, showed that levels of IL-1β and IL-18 in the MPTP treatment were significantly increased, while these changes could be reversed by Sal group. To investigate the anti-pyroptosis mechanism of Sal in MPTP-induced PC-12 cells, we conducted western blotting and immunofluorescence. The results of western blotting demonstrated the up-regulation of TLR4, myD88, p-IκBα, p-NF-κB, TXNIP, NLRP3, ASC, cleaved Caspase-1, cleaved GSDMD, IL-1β and IL-18 in MPTP group, while the Sal treatment groups effectively inhibited these alterations (Figure 7A). In agreement with the above, the levels of TLR4 and TXNIP in Sal (50 μM) treatment group were significantly lower than those in MPTP group by immunofluorescence (Figure 7B). These results revealed that Sal suppressed pyroptosis by inhibiting the TLR4/MyD88/NF-κB and TXNIP/NLRP3/Caspase-1 signaling pathways in MPTP-induced PC-12 cells.

### TLR4 plays a vital role in MPTP-induced pyroptosis

**Figure 8:**
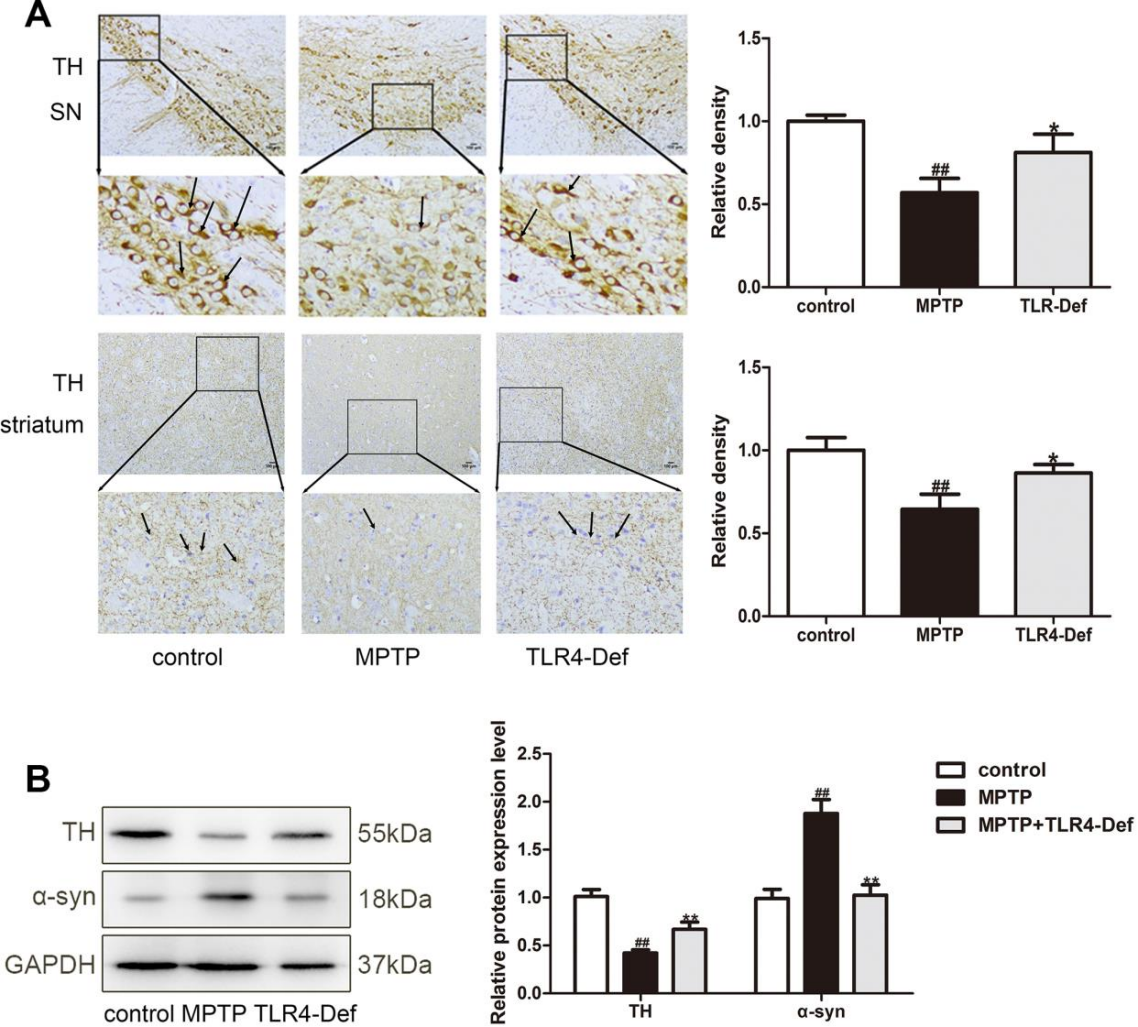
**TLR4 plays a vital role in MPTP-induced pyroptosis.** (**A**) TLR4-Def group inhibited MPTP-induced the reduction of TH-positive neurons in SN and striatum by immunochemical staining (n = 3). Original magnification: x200. (**B**) Western blotting was performed to determine the expressions of TH and α-syn in SN and striatum of TLR4-Def PD mice (n = 3). All data are represented as mean ± SD. ^#^ P < 0.05, ^##^ P < 0.01 vs. control group, *P < 0.05, **P < 0.01 vs MPTP group.

**Figure 9:**
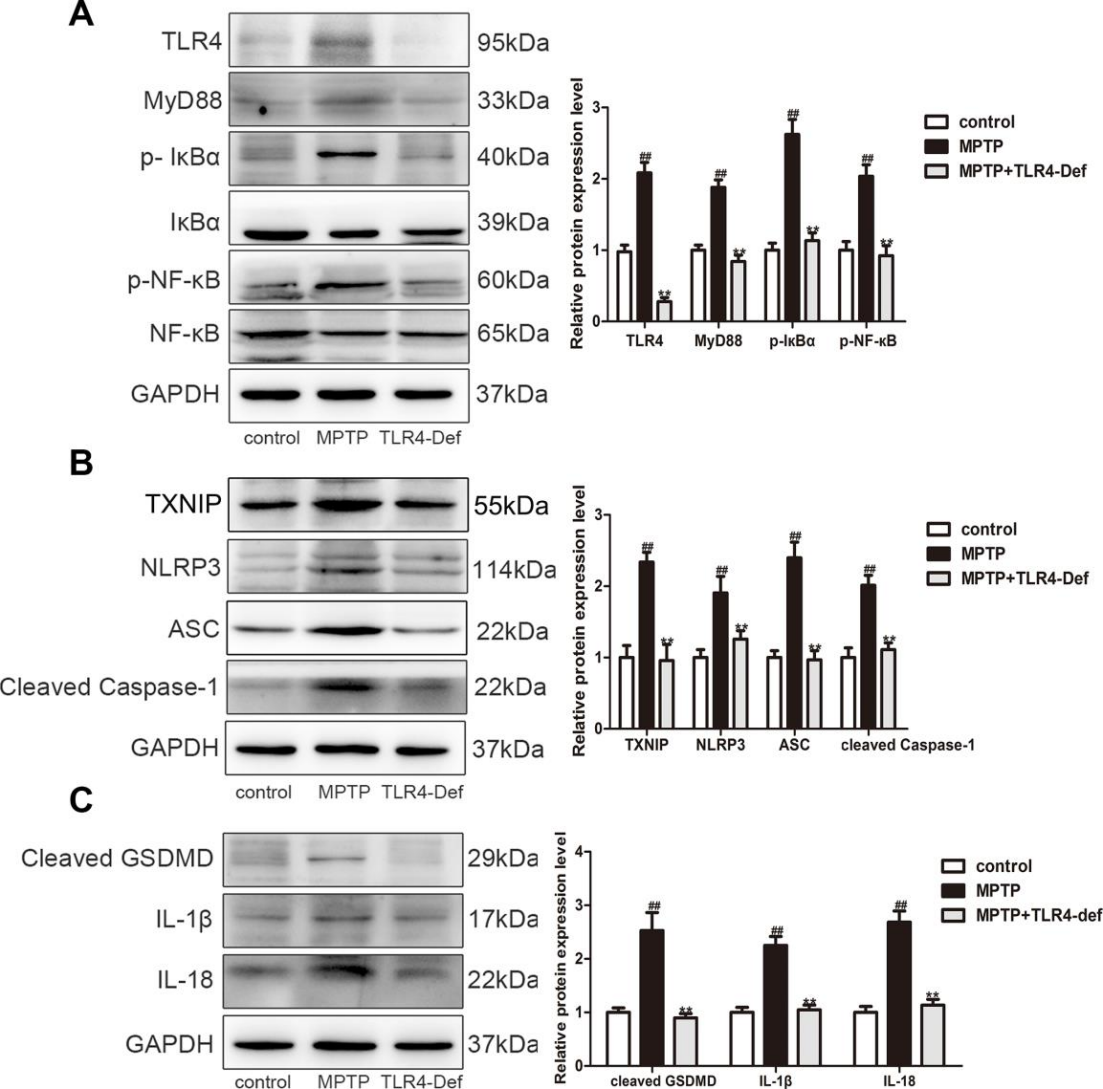
**TLR4 aggravated pyroptosis by activating the TLR4/MyD88/NF-κB and NLRP3/ASC/Caspase-1 signaling pathways.** (**A**–**C**) Western blotting was performed to determine the expressions of TLR4, MyD88, p-IкBα, p-NF-кB, TXNIP, NLRP3, ASC, cleaved caspase-1, cleaved GSDMD, IL-1β and IL-18 in SN and striatum of TLR4-Def PD mice (n = 3). All data are represented as mean ± SD. ^#^ P < 0.05, ^##^ P < 0.01 vs. control group, *P < 0.05, **P < 0.01 vs MPTP group.

We further used C57BL/10ScNJ mice (a TLR4-deficient mice, TLR4-Def) to investigate whether TLR4 plays a vital role in pyroptosis in MPTP-induced PD mice. In TLR4-Def group displayed the increased number of TH-positive neurons in SN and striatum in MPTP-induced mice by immunohistochemistry (Figure 8A). TLR4-Def group also increased TH protein and decreased α-syn in SN and striatum of PD mice (Figure 8B). Preliminary results showed that TLR4 plays a key role in the pathogenesis of PD. Previous studies have shown that pyroptosis is crucial in PD, and we further verified the specific mechanisms in it. Our results demonstrated that TLR4 plays a vital role in MPTP-induced pyroptosis. The main mechanisms inhibited MyD88, p-IκBα, and p-NF-κB protein expressions (Figure 9A). It was indicated that the production of IL-1β, IL-18 and NLRP3 is inhibited by TLR4/MyD88/NF-κB signaling pathways. At the same time, we also detected NLRP3-depend pyroptosis-related signaling pathway. It is interestingly that TLR4-Def group also inhibited MPTP-induced increased of TXNIP, NLRP3, ASC, cleaved Caspase-1 and cleaved GSDMD protein expressions (Figure 9B). The result showed that TLR4-Def group inhibited MPTP-induced pyroptosis related indicators, including cleaved GSDMD, IL-1β and IL-18 in PD mice (Figure 9C). The above experiments further indicated that TLR4 plays a vital role in MPTP-induced pyroptosis.

### Sal inhibited LPS-induced BV2 cells inflammation response via TLR4/MyD88/ NF-κB signaling pathways

**Figure 10:**
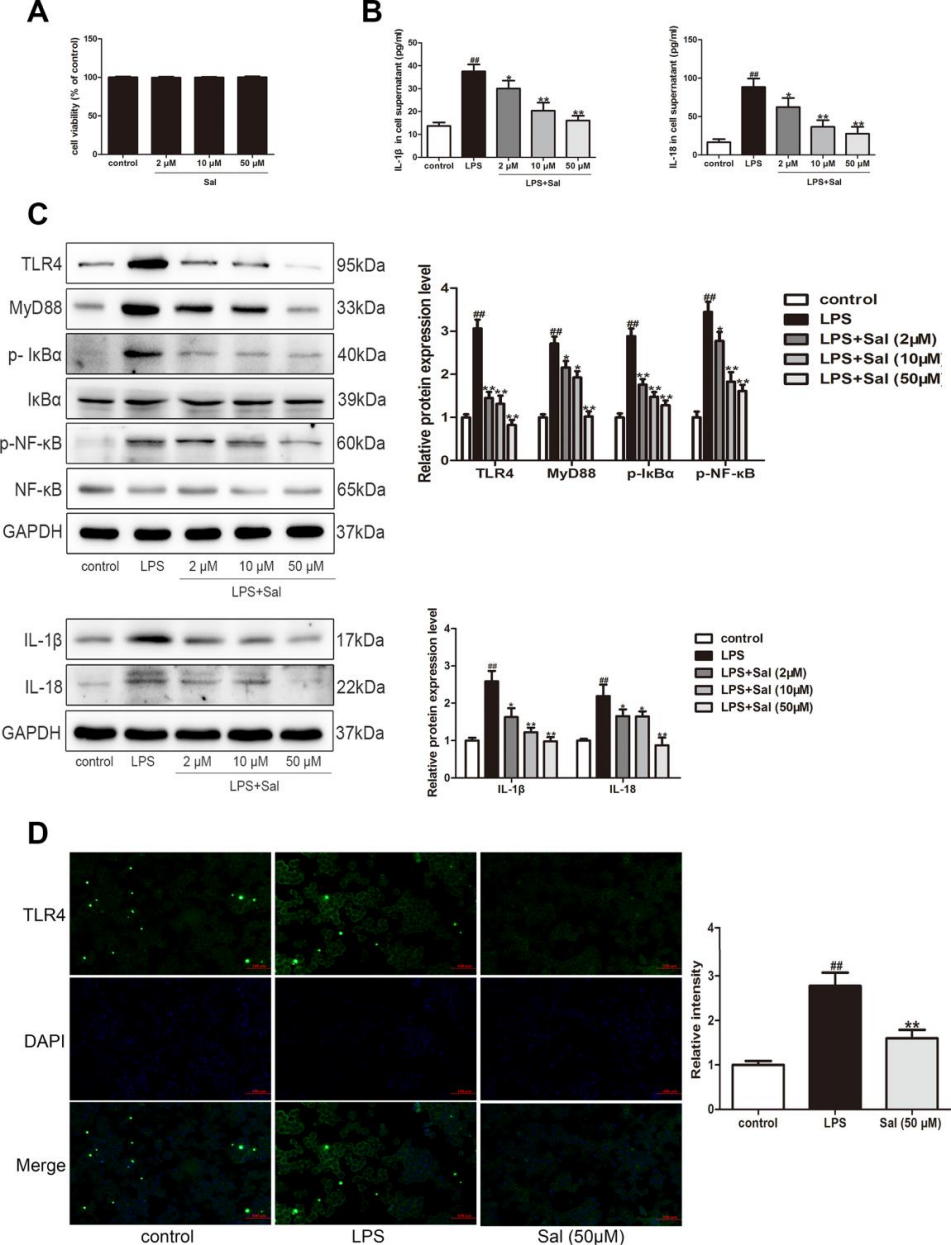
**Sal inhibited LPS-induced BV2 cells inflammation response via TLR4/MyD88/ NF-κB signaling pathways.** (**A**) Effect of different doses of Sal on BV2 cell viability. BV2 cells (1 x 10^4^ cells/well) were exposed to different concentrations of Sal (2, 10, 50 μM) for 24 h. The cell viability of BV2 was measured by CCK8 assay. (**B**) The levels of IL-1β and IL-18 in the supernatant of BV2 cells were determined by ELISA kits (n = 6). BV2 cell were treated with Sal (2, 10, 50 μM) for 2 h, followed by stimulation with LPS (100 ng/ml) for 24 h. (**C**) Western blotting was performed to determine the expressions of TLR4, MyD88, p-IкBα, p-NF-кB in LPS-induced BV2 cells. The cells were incubated with Sal (2, 10, 50 μM) for 2 h, followed by stimulation with LPS (100 ng/ml) for 30 min. Western blotting was performed to determine the expression of IL-1β and IL-18 in LPS-induced BV2 cells. The cells were incubated with Sal (2, 10, 50 μM) for 2 h, followed by stimulation with LPS (100 ng/ml) for 24 h. (**D**) Immunofluorescence staining of TLR4 in BV2 cells. The cells were incubated with Sal (50 μM) for 2 h, followed by stimulation with LPS (100 ng/ml) for 30 min. Original magnification: x200. All data are represented as mean ± SD. ^#^ P < 0.05, ^##^ P < 0.01 vs. control group, *P < 0.05, **P < 0.01 vs LPS group.

Microglia cells are thought to link between the immune and central inflammation response [37]. To investigate the role of Sal on TLR4 in microglia cells, we used lipopolysaccharide (LPS, a TLR4 receptor agonist) to stimulate the BV2 cells. Firstly, we assessed cytotoxicity of Sal on BV2 cells. The results showed that Sal (2, 10, 50 μM) treatment did not affect the viability of BV2 cells (Figure 10A). The results demonstrated that TLR4 induced the increase of IL-1β and IL-18, and then we further tested whether Sal could improve LPS-induced BV2 cell inflammation response. Our results showed that Sal inhibited LPS-induced BV2 cells increased IL-1β and IL-18 in the supernatant (Figure 10B). Sal also suppressed IL-1β and IL-18 protein expression in BV2 cells (Figure 10C), and then Further suppressed IL-1β and IL-18-induced pyroptosis. TLR4 plays a vital role in PD mice, so we further verified whether Sal inhibited TLR4 related signaling pathways. The results showed that Sal inhibited the increase of TLR4, MyD88, p-IκBα and p-NF-κB in LPS-induced BV2 cell (Figure 10C). Further study indicated that Sal (50 μM) inhibited TLR4 in LPS-induced BV2 cells by immunofluorescence (Figure 10D). As expected, these results suggested that Sal suppressed inflammatory-related pyroptosis cytokines IL-1β and IL-18 by inhibiting the TLR4/MyD88/NF-κB signaling pathways.

## DISCUSSION

**Figure 11:**
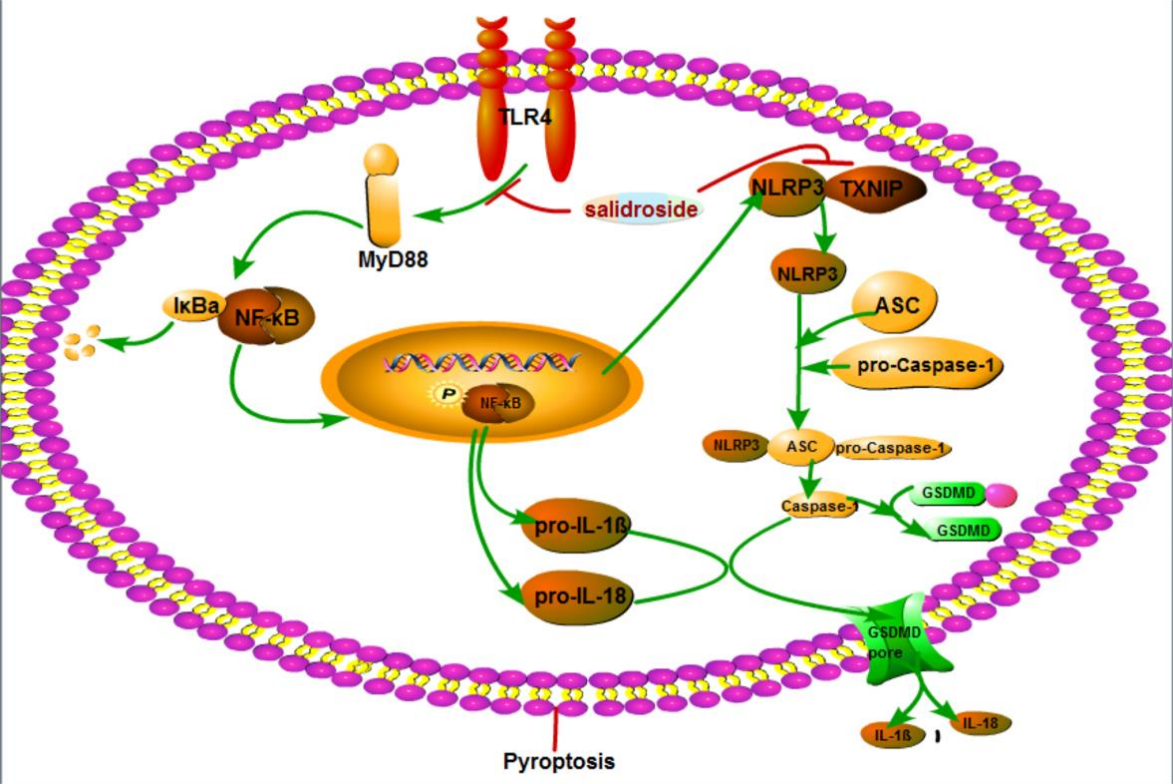
**Schematic mechanism illustration of Sal ameliorates PD by inhibiting the NLRP3-dependent pyroptosis.** The main mechanisms are as follows: (1) Sal indirectly reducing the production of NLRP3, pro-IL-1β and pro-IL-18 by inhibiting TLR4/MyD88/NF-κB signaling pathways; (2) Sal directly suppressing pyroptosis through inhibiting TXNIP/NLRP3/caspase-1 signaling pathways. These results indicated that inhibiting pyroptosis or administration of Sal could be a novel therapeutic strategy for PD.

Accumulating evidence suggests that Sal has many pharmacological activities. In particular, Sal exhibits anti-inflammatory and anti-oxidant, both *in vitro* and *in vivo*. In present study, we reveal that Sal partially inhibits DA neurons pyroptosis and its potential mechanisms. The new findings are as follows: (1) Pyroptosis plays an important role in the development of PD. (2) Sal ameliorates PD by inhibiting pyroptosis *in vitro* and *in vivo*. (3) Sal inhibits the pyroptosis by the following two aspects: 1) indirectly reducing the production of NLRP3, pro-IL-1β and pro-IL-18 by inhibiting TLR4/MyD88/NF-κB signaling pathways, 2) directly suppressing pyroptosis through inhibiting TXNIP/NLRP3/Caspase-1 signaling pathways, as illustrated in Figure 11.

PD is a common age-related neurodegenerative disease and typically characterized by loss of dopaminergic neurons [6–8]. The number of DA neurons progressively reduced at least 50% of SN DA neurons and more than 80% of striatum DA neurons in PD patients [38]. MPTP, a selective toxin for DA neurons, could induce humans and non-human primates to parkinson symptoms [39]. Therefore, neurotoxin MPTP has been one of the most extensive recognized drugs to induce PD model *in vivo* and *in vitro* [39, 40]. PC-12 cells, derived from a pheochromocytoms of rat adrenal medulla, exposure to nerve growth factor (NGF) to induce neuronal differentiation [41]. PC-12 cells could differentiate to dopaminergic neuron-like cells, and then secret dopamine, norepinephrine and acetylcholine [41]. So it has been widely used as a PD model *in vitro* [41–43]. Therefore, MPTP-induced dopaminergic neuron loss in C57BL/6 mice and MPTP-induced PC-12 cell injury were used as our experimental models *in vitro* and *in vivo*. The results showed that Sal effectively improved MPTP-induced behavioral disorder. TH is a rate-limiting enzyme in DA biosynthesis and is closely related to the development of PD. Our studies demonstrated that MPTP-treated mice showed a 54% decrease in TH-positive neurons, which is consistent with previous studies [9, 44] and Sal could increase TH-positive cells in SN and striatum. In addition, the defects of protein degradation are common in neurodegenerative pathologies [45]. It is well known that the excessive accumulation of α-syn is another typical pathological features of PD [4, 5]. Previous study showed that α-syn aggregates might activate NLRP3 inflammasome through microglia endocytosis to participant in the pathogenesis of PD [46]. Our results indicated that Sal treatment significantly decreased α-syn accumulation *in vivo* and *in vitro*. These results suggested that Sal could be a promising agent for PD.

Central nervous system (CNS) immune activation and then neuro-inflammation occur in various neurodegenerative diseases [47]. Although the precise pathogenesis of PD still remains elusive, many studies suggested that inflammation and oxidative stress are crucial in PD pathogenesis [11, 47]. As previously reported, inflammatory factors such as IL-6, IL-1β and IL-18 were notably increased in PD patients [28, 29]. Indeed, IL-1β and IL-18 are critically involved in the development of PD [29, 48]. Previous studies demonstrated that Sal plays a protective role in anti-inflammatory in neurodegenerative diseases [49]. Our result showed that Sal significantly counteracted the MPTP-induced increase levels of IL-1β and IL-18 *in vivo* and *in vitro*. The secretion of IL-1β and IL-18 is mainly regulated by NLRP3 inflammasome [50]. As cytosolic multi-protein complexes, inflammasome can be divided into subtypes based on the different combinations of molecules, and the main is NLRP3 inflammasome. NLRP3 inflammasome, including NLRP3, ASC and Caspase-1, is currently the most fully characterized inflammasome [51]. NLRP3 inflammasome activation has been confirmed in neurodegenerative diseases, including PD and Alzheimer’s disease (AD) [52, 53]. Moreover, NLRP3 KO mice exhibited the decrease of IL-1β protein in the brain and alleviated inflammation responses [54]. In addition, Oxidative stress is also a critical in the pathogenesis of PD [55]. Thioredoxin-interacting protein (TXNIP), an endogenous inhibitor of thioreedoxin (TRX), is associated with activation of NLRP3 inflammasome [56, 57]. We found that Sal effectively reduced TXNIP *in vivo* and *in vitro*. The activation of NLRP3 can not only promote caspase-1 maturation but also promote the secretion of IL-1β and IL-18 and then induce pytoptosis [58]. As an inflammasome-mediated programmed cell death, pyroptosis plays a pivotal role in maintaining homeostasis and in removing unnecessary cells [59]. Actived NLRP3-dependent pyroptosis can further increase the release of IL-1β and IL-18 and promote inflammatory responses [60]. The activity of caspase-1 is closely regulated by multi-protein complexes called ‘NLRP3 inflammasome’ and activated caspase-1 could regulate the cleavage of the inactive precursor pro-IL-1β and pro-IL-18 to active IL-1β and IL-18 [61, 62]. As a pyroptosis executive protein, GSDMD oligomerization is initiated by caspase-1-mediated removal of C-terminal inhibitory domain, which activates GSDMD [21]. The matured IL-1β and IL-18 are secreted extracellular by GSDMD pore [21, 30] to exert inflammatory effect, and further induce DA neurons damage. In our study, we found that Sal inhibited IL-1β, IL-18 and cleaved GSDMD levels *in vivo* and *in vitro*. Its main mechanism is directly inhibiting TXNIP/NLRP3/Caspase-1 dependent pyroptosis.

TLR4, an important member of the TLRs family, is involved in various neurodegenerative diseases, including Alzheimer's disease (AD), Parkinson's disease (PD), Huntington's disease (HD), amyotrophic lateral sclerosis (ALS) and so on [63–65]. TLR4 combines myeloid differentiation primary response protein 88 (MyD88) proteins, activates nuclear factor-κB (NF-κB) and then stimulates to cause a cascade of inflammatory responses [66]. Activated NF-κB transported from cytoplasm to nucleus and then promoted the secretion of pro-IL-1β, pro-IL-18 and NLRP3, which are crucial for regulation of pyroptosis [67, 68]. Our results displayed that Sal significantly reduced the expressions of TLR4, MyD88, p-NF-κB *in vitro and in vivo*. To further study the role of TLR4 in NLRP3-dependent pyroptosis in PD, we used TLR4 deficient mice (C57BL/10ScNJ, TLR4-Def) to investigate it. The results showed that TLR4-Def mice reversed pathology in PD model, and reduced NLRP3 and α-syn aggregates, which preserved DA neurons loss and associated neurologic deficit. These results suggested TLR4 plays an important role in PD and increases NLRP3-dependent pyroptosis.

Microglia, the first line of defense when injury or diseases occur, are the innate immune cells of the central nervous system [69, 70]. Microglia activation in the brain of PD patients results in non-autonomous cell processes and even DA neurons degeneration [71]. The activation of microglia can secrete many neurotransmitters and pro-inflammatory cytokines such as IL-1β and IL-18, which not only regulate the inflammatory response, but also directly and indirectly damage neurons [72]. Toll-like receptors (TLRs) have been regarded as the primary innate immune receptors that can be activated by endogenous danger factors and then induced inflammation responses in PD [73]. In this study, we explored the other potential mechanisms of neuro-inflammation in PD by LPS-stimulated BV2 microglia cells. LPS can activate NF-κB to transport from cytoplasm to nucleus by TLR4/MyD88/NF-κB signaling pathways and then up-regulate downstream inflammatory factors and promote the secretion of IL-1β and IL-18. Our results displayed that Sal could reduce the release of IL-1β and IL-18 by inhibiting the TLR4/MyD88/NF-κB signaling pathways.

In conclusion, our data strongly support that pyroptosis plays an essential role in development of PD and Sal ameliorates DA neuronal damage by suppressing NLRP3-dependent pyroptosis. These findings indicated that inhibiting pyroptosis or administration of Sal could be a novel therapeutic strategy for PD.

## MATERIALS AND METHODS

### Main reagents and kits

Salidroside was provided by the Second Medical University (Shanghai, China; purity > 99%). MPTP-HCl was purchased from MedChemExpress (New Jersey, USA). Lipopolysaccharide (LPS) was purchased from Sigma Aldrich (St. Louis, USA). Enzyme-linked immunosorbent assay (ELISA) kits of interleukin (IL)-18 and IL-1β were purchased from Elabscience (Wuhan, China). The primary antibodies against MyD88, p-IκBα, IκBα, NF-κB, ASC, cleaved-Caspase-1, GSDMD, α-Synuclein, TH, TXNIP and GAPDH were purchased from Cell Signaling Technology (Danvers, MA, USA). The anti-p-NF-κB, TXNIP, NLRP3, IL-1β and IL-18 primary antibodies were produced by Abcam (Cambridge, UK). The anti-TLR4 primary antibody was purchased from Santa Cruz Biotechnology (Santa Cruz, CA). The antibodies are listed in Supplementary Table 1 and the critical chemicals are listed in Supplementary Table 2.

### Animals and experimental design

Eight-week-old male C57BL/6 mice weighing 22-25g were supplied by the Jiangning Qinglongshan Animal Cultivation Farm (Nanjing, China) and were acclimated for 7 days prior to the experiments under a standard laboratory animal facility (25°C, 12 h light/dark cycle) with food and water ad libitum. Forty male mice were randomly assigned to four groups (ten mice per group): (1) Control group, (2) MPTP group, (3) MPTP + Sal (40 mg/kg) group, (4) MPTP + Sal (80 mg/kg) group. The mice were injected with MPTP (30mg/kg, dissolved in normal saline, intraperitoneal i.p.) for 5 days, while the control group mice were injected with the same amount of normal saline (i.p.). Mice were given Sal (40 and 80 mg/kg) at corresponding dose by daily intragastric gavage (i.g.), while the control group mice were given identical volumes of purity water. The mice were sacrificed for brain tissue after behavioral tests.

Eight-week-old male C57BL/10ScNJ (TLR4-deficient) mice weighing 22-25g were supplied Model Animal Research Center of Nanjing University and were acclimated for 7 days prior to the experiments. The mice were assigned to three groups (ten mice per group): (1) Wild type (control) group, (2) MPTP group, (3) MPTP+TLR4-Def (TLR4-Def) group. The mice were injected with MPTP (30 mg/kg, dissolved in normal saline, intraperitoneal, i.p.) for 5 days, while the control group were injected with the same amount of normal saline (i.p.).

### Behavioral tests

### Pole test

The pole test was performed according to previously published protocols [74]. Mice were adapted to the environment for 3 days prior to the testing and performed 1 day after treatments. During the test, mice whose heads faced upwards were placed on the top of a rough surfaced pole (1 cm in diameter and 55 cm in height) and climbed down the pole. The time required for mice to turn completely downwards (T1) and to climb down the pole (T2) were recorded. It is required for us to re-test when the mice stoped halfway or climbed reverse. The experiments were performed by examiners blinded to each group.

### Open-field test

Mice were placed in a white square arena(45×45×60 cm), and mice behavior on the arena were continuously recorded for 3 minutes with a video camera (Sony CCD IRIS; Sony, Tokyo, Japan) located above the arena. Results of the open-field test were analyzed with ANY-Maze animal behavior analysis system (Zhongshidichuang Science and Technology Developmant Co., Ltd.). We monitored the tracks of mice, distance, average velocity, during 3-minute open-field test. The open-field arena was cleaned with 70% ethyl alcohol and was permitted to dry between tests. The experiments were performed by examiners blinded to each group.

### Cell culture

Rat adrenal pheochromocytoma cell lines (PC-12) were obtained from the Cell Bank of the Chinese Academy of Sciences (Shanghai, China) and BV2 cell lines was purchased from the American Type Culture Collection. Both cultured in Dulbecco's modified Eagle's medium (DMEM, high glucose, NanJing KeyGen Biotech Co., Ltd.) containing 10 % fetal bovine serum (FBS, Gibco), penicillin (100 IU/ml) and streptomycin (100 μg/ml). Cells were cultured in a humidified incubator with 5% CO_2_ at 37°C and medium was replaced every 2-3 days.

The PC-12 cells were adjusted to 2×10^5^cell/well and were seeded in a 6-well plate. The cells were incubated with Sal (2, 10, 50 μM) for 2 h, followed by stimulating with MPTP (500 μM) for 24 h. Finally, all the cells were collected for the various analyses.

The BV2 cells were seeded in a 6-well plate at a density of 2×10^5^cell/well. Then, cells were incubated with Sal (2, 10, 50 μM) for 2 h, followed by stimulating with LPS (100 ng/ml) for 30 min. Finally, all the cells were then collected for the various analyses.

### Cell viability assay

The PC-12 cells (1×10^4^cells/well) and BV2 cells (1×10^4^cells/well) were seeded in 96-well plates and cell viability was measured by the Cell Counting Kit-8 (CCK-8, Beyotime Biotechnology, Nantong, China). The data were assessed as the percentage of the average absorbance to the control group. Cell viability (%) = (A Treat/A Control)×100 %.

### Inflammatory cytokines levels in brain and cell supernatant

The concentrations of IL-1β and IL-18 in brain and cell supernatant were determined by enzyme-linked immunosorbent assay (ELISA) kits according to the manufacturer's instructions (Elabscience, China).

### Immunohistochemistry staining

SNpc and striatum were processed for Immunohis-tochemistry (IHC) analysis**.** IHC was performed as described in previous reports with minor modifications [53]. Briefly, the mice were perfused with 4% paraformaldehyde (PFA). Subsequently, we removed the brains and fixed in 4% PFA for 48h and then embedded in paraffin and sliced into 5 μm thick sections. Three mice per group (one mice for two sections) for IHC, totally six section of SNpc and striatum was processed for IHC. The sections were were dewaxed by xylene and hydrated in graded ethanol, then micro-waved in sodium citrate buffer. The endogenous peroxidase was blocked with 3% hydrogen peroxide for 30 min. Each sample was blocked with 5% goat serum for 30 min and then treated with primary antibodies TH (Cell Signaling Technology, #5884, 1:300), α-syn (Cell Signaling Technology, #4179, 1:200), TXNIP (Abcam, ab188865, 1:200) and TLR4 (Santa cruz, sc-293072, 1:50) at 4°C overnight. On the second day, the sections were washed and incubated with the goat anti-rabbit IgG (the first three primary antibodies) and the goat anti-mouse IgG (the last primary antibodies) secondary antibodies for 30 min. Then, the sections were stained with 3,3- diaminobenzidine (DAB) and counterstained with hematoxylin. After dehydrating and drying, they were mounted with neutral gum. Images were collected using an inverted fluorescent microscope (Nikon, Ts2R, Japan). For the densitometric analyses, the percentage of positive staining (brown staining) was measured by Image J.

### Immunofluorescence staining

The expressions of TLR4, TXNIP in the PC-12 cells and The expression of TLR4 in BV2 cells were evaluated by immunofluorescence. Briefly, the cells were fixed with 4% paraformaldehyde for 30 min and washed three times in PBS (5 min/time). Subsequently, the cells were punched with 0.3% Triton X100 for 15 min and blocked with 5% BSA for 2 h, The primary antibodies TLR4 (Santa cruz, sc-293072, 1:50) and TXNIP (Cell Signaling Technology, #14715, 1:50) were incubated at 4°C overnight. After washing with phosphate buffer saline (PBS), the cells were incubated with a fluorescence-conjugated antibody (1:400) for 2 h at room temperature. After washing three times with PBS (5 min/time) and then with 4',6-diamidino-2-phenylindole (DAPI) for 5 min. Cells were observed and captured with inverted fluorescent microscope (Nikon, Ts2R, Japan).

### Western blot

Substantia nigra and striatum, PC-12 and BV2 cells were homogenate in an ice-cold RIPA buffer containing 1 mM phenylmethyl- sulfonyl fluoride (PMSF). Lysates were incubated on ice for 20 min and then the samples were centrifuged at 12,000×g for 15 min at 4°C. The supernatant was collected and then the total protein was detected by BCA protein assay kit (Beyotime Biotechnology, Nantong, China). The proteins were separated by SDS-polyacrylamide gelelectrophoresis (SDS-PAGE) and transferred to polyvinylidene fluoride (PVDF) membranes. The membranes were incubated in 5% skim dried milk for 2 h at room temperature, and then incubated with primary antibodies TH (Cell Signaling Technology, #5884,1:1000), α-syn (Cell Signaling Technology, #4179, 1:1000), TLR4 (Santa cruz, sc-293072, 1:200), MyD88 (Cell Signaling Technology, #4283, 1:1000), p-IκBα (Cell Signaling Technology, #2859, 1:1000), IκBα (Cell Signaling Technology, #4814, 1:1000), NF-κB (Cell Signaling Technology, #8242, 1:1000), p-NF-κB (Abcam, ab86299, 1:1000), TXNIP (Cell Signaling Technology, #14715, 1:1000), NLRP3 (Abcam, ab214185, 1:1000), ASC (Cell Signaling Technology, #67824, 1:1000), cleaved Caspase-1 (Cell Signaling Technology, #89332, 1:1000), IL-1β (Abcam, ab9722, 1:1000), IL-18 (Abcam, ab207323, ab191860, 1:1000) and GAPDH (Cell Signaling Technology, #2118, 1:1000) overnight at 4°C. After washing with TBST for three times (5 min/time), the membranes were incubated with the second antibodies (1:1000) at room temperature for 2 h. The membranes were washed and then visualized using an ECL advanced kit and detected with a gel imaging system (Tanon Science and Technology Co., Ltd., China).

### Statistical analysis

All data are expressed as the mean ± standard deviation (SD). The differences between the different groups were analyzed by one-way analysis of variance (ANOVA), followed by Tukey's multiple comparison test. *P <0.05* was considered statistically significant.

## Supplementary Material

Supplementary Tables
