# Supplementary material for: Salidroside ameliorates Parkinson's disease by inhibiting NLRP3-dependent pyroptosis
Source: Aging (Albany NY). 2020 May 19;12(10):9405–26. doi: 10.18632/aging.103215 (PMC7288953; doi:10.18632/aging.103215)
Supplement: Supplementary Tables [file aging-12-103215-s001..pdf]

SUPPLEMENTARY TABLES

Supplementary Table 1. Antibodies.

Protein name	Company	Identifier
Mouse monoclonal anti-TLR4	Santa cruz	sc-293072
Rabbit monoclonal anti- MyD88	Cell Signaling Technology	#4283
Rabbit monoclonal anti- p- IκBα	Cell Signaling Technology	#2859
Mouse monoclonal anti- IκBα	Cell Signaling Technology	#4814
Rabbit monoclonal anti- NF-κB p65	Cell Signaling Technology	#8242
Rabbit monoclonal anti-ASC	Cell Signaling Technology	#67824
Rabbit monoclonal anti-Cleaved- caspase-1	Cell Signaling Technology	#89332
Rabbit polyclonal anti- Gasdermin D	Cell Signaling Technology	#93709
Rabbit monoclonal anti- Tyrosine Hydroxylase	Cell Signaling Technology	#58844
Rabbit monoclonal anti- Alpha-Synuclein	Cell Signaling Technology	#4179
Rabbit monoclonal anti-TXNIP	Cell Signaling Technology	#14715
Rabbit polyclonal anti- p-NF-κB p65	Abcam	ab86299
Rabbit polyclonal anti-NLRP3	Abcam	ab214185
Rabbit polyclonal anti- IL-1β	Abcam	ab9722
Rabbit polyclonal anti-IL-18	Abcam	ab191860
Rabbit monoclonal anti-IL-18	Abcam	ab207323
Rabbit monoclonal anti-TXNIP	Abcam	ab188865
Anti-rabbit IgG, HRP-linked Antibody	Cell Signaling Technology	#7074
Anti-mouse IgG, HRP-linked Antibody	Cell Signaling Technology	#7076
Anti-mouse IgG (H+L) Alexa Fluor(R) 488	Cell Signaling Technology	#4408
Anti- rabbit IgG (H+L) Alexa Fluor(R) 488	Thermo Fisher Scientific	A11008

Supplementary Table 2. Critical chemicals and commercial assays.

Reagents	Source	Identifier
MPTP hydrochloride	MedChem Express	23007-85-4
Lipopolysaccharide	Sigma-Aldrich	L2880
Dulbecco's modified Eagle medium (High Glucose)	NanJing KeyGen	KGM12800-500
Fetal bovine serum	Gibco	1600044
Trypsin-EDTA (0.25 %)	Gibco	25200072
Enhanced Cell Counting Kit-8	Beyotime Biotechnology	C0042
Mouse IL-1β Elisa Kit	Elabscience	E-EL-M0037
Rat IL-1β Elisa Kit	Elabscience	E-EL-R0012
Mouse IL-18 Elisa Kit	Elabscience	E-EL-M0730
Rat IL-18 Elisa Kit	Elabscience	E-EL-R0567
